# Biological characterization and clinical application of tRF in cognitive impairment associated with white matter injury after chronic hypoperfusion

**DOI:** 10.1002/alz.094918

**Published:** 2025-01-09

**Authors:** Min Fang

**Affiliations:** ^1^ St. Luke’s Hospital, Shanghai, Shanghai China

## Abstract

**Background:**

Recent studies have revealed that tRNA‐derived fragments(tRFs)are closely associated with the immunoinflammatory process of brain injury after stroke.

**Method:**

Firstly, 3 patients with WMI and the same number of healthy volunteers' peripheral blood in the control group were collected. EVS in the subjects' peripheral plasma was isolated and purified, and tRFs high‐throughput sequencing was performed. Subsequently, we verified significant differential expression of tRFs in sera from different oxygen glucose deprivation (OGD) cell lines and WMI patients. Next, possible tRF‐bound proteins were detected by RNA pull down combined with LC‐MS/MS targeted quantitative mass spectrometry. Finally, we collected serum samples from patients with WMI, including 20 each of cognitively normal and WMI‐associated cognitive impairment patients, analysed the relationship between significantly different tRFs and clinical indicators, and analysed the diagnostic efficacy of tRFs for WML‐associated cognitive impairment by ROC curves.

**Result:**

1. The expression level of 3'Val‐TAC tRF was significantly higher in the serum of patients with WMI compared with controls; 2. 3'Val‐TAC tRF was not only significantly up‐regulated in OGD neuronal cells, but also was significantly highly expressed in the sera of patients with WMI; 3. 3'Val‐TAC tRF originated from mitochondrial tRNA, which belongs to tRF‐3b. A total of 86 proteins bound to the 3'Val‐TAC tRF compared to the negative control. Given the 87% peptide coverage of USP30 in the mass spectrometry results, it is highly suggestive that 3'Val‐TAC tRF is able to bind to USP30;4. The expression level of 3'Val‐TAC tRF was negatively correlated with the MoCA score (r = 0.376, P = 0.017). ROC curve analysis showed that 3'Val‐TAC tRF expression level had a high diagnostic value for WMI‐related cognitive impairment (AUC 0.835, P<0.001).

**Conclusion:**

3'Val‐TAC tRF should be a small RNA that is highly expressed in WMI patients and induces WMI‐related cognitive impairment.

